# Supplementary material for: Norepinephrine Leads to More Cardiopulmonary Toxicities than Epinephrine by Catecholamine Overdose in Rats
Source: Toxics. 2020 Sep 16;8(3):69. doi: 10.3390/toxics8030069 (PMC7560392; doi:10.3390/toxics8030069)
Supplement: Supplementary file 1 [file toxics-08-00069-s001.pdf]

# Supplementary Materials: Norepinephrine Leads to More Cardiopulmonary Toxicities than Epinephrine by Catecholamine Overdose in Rats

**Wen-Hsien Lu, Hsin-Hung Chen, Bo-Hau Chen, Jui-Chen Lee, Chi-Cheng Lai, Che-Hsing Li and Ching-Jiunn Tseng**

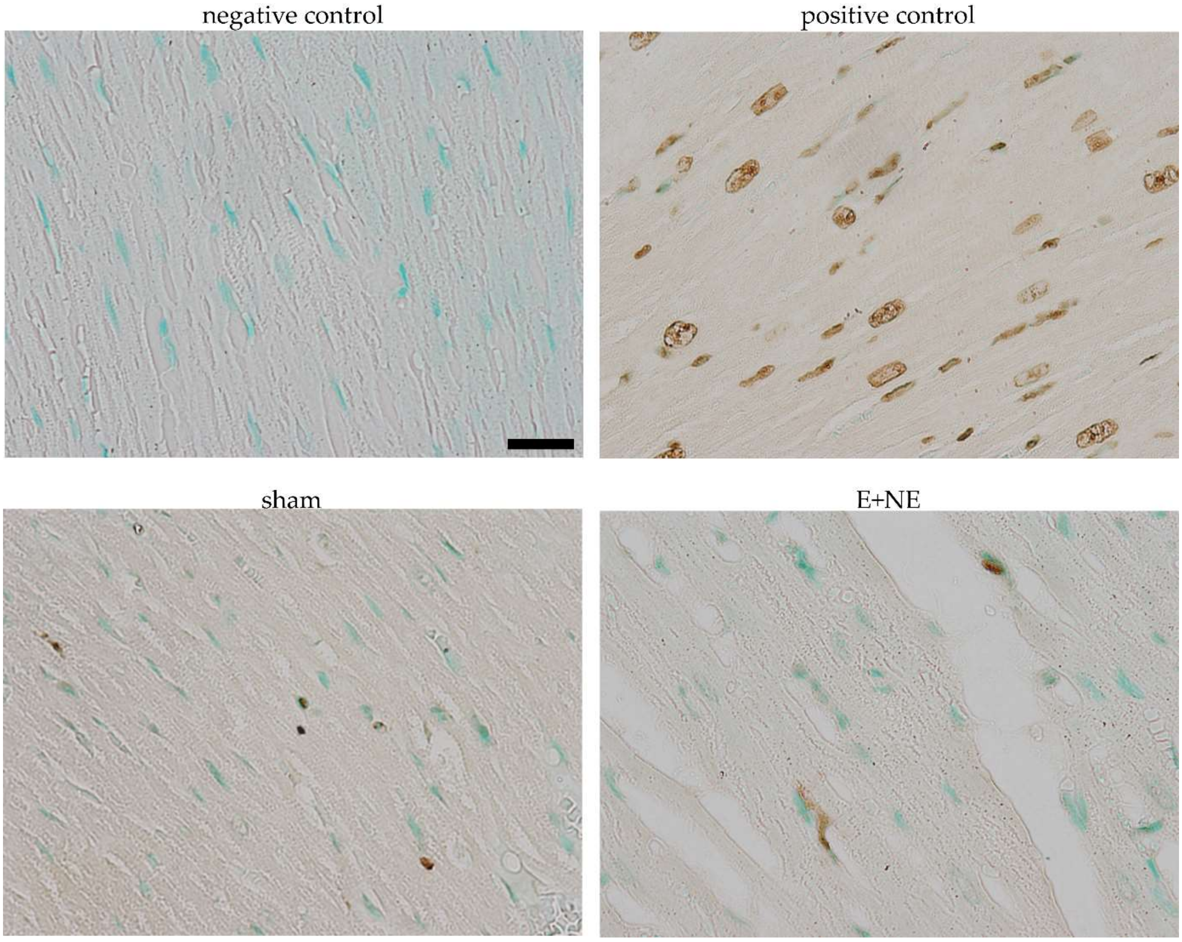


**Figure S1.** Apoptosis induced by catecholamine overdose through Terminal deoxynucleotidyl transferase dUTP nick end labeling assay. Sham, 0.9% saline injection group; E+NE, epinephrine and norepinephrine injection group.

**Table S1.** The echocardiographic parameters of the 4 group at 0-h and 6-h post-intravenous continuous infusion of epinephrine and/or norepinephrine.

| Parameters | Echocardiographic parameters | | | | | | | | | | | |
|---|---|---|---|---|---|---|---|---|---|---|---|---|
| | sham ($n$ = 3) | | | E ($n$ = 6) | | | NE ($n$ = 6) | | | E+NE ($n$ = 6) | | |
| | 0 h (Mean±SD) | 6 h (Mean±SD) | $p$ value | 0 h (Mean±SD) | 6 h (Mean±SD) | $p$ value | 0 h (Mean±SD) | 6 h (Mean±SD) | $p$ value | 0 h (Mean±SD) | 6 h (Mean±SD) | $p$ value |
| Body weight (g) | 378.7±10.97 | | | 367.17 ± 12.56 | | | 372.5 ± 15.53 | | | 378.67 ± 3.95 | | |
| HR (bpm) | 355.12 ± 42.93 | 368.12 ± 35.14 | 0.273 | 333.92 ± 40.84 | 487.27 ± 82.88* | 0.028 | 302.69 ± 19.90 | 524.91 ± 51.55* | 0.028 | 329.76 ± 22.59 | 457.16 ± 73.25* | 0.028 |
| IVSd (mm) | 1.83 ± 0.14 | 1.80 ±0.04 | 0.715 | 1.88 ± 0.22 | 2.51 ± 0.56 | 0.046 | 1.73 ± 0.06 | 3.27 ± 0.24*† | 0.028 | 2.05 ± 0.31 | 3.24 ± 0.33* | 0.028 |
| LVIDd (mm) | 7.090 ± 0.44 | 7.18 ± 0.16 | 0.273 | 7.37 ± 0.30 | 5.64 ± 1.36 | 0.046 | 7.15 ± 0.48 | 4.16 ± 1.07* | 0.028 | 7.04 ± 0.48 | 3.20 ± 0.44*† | 0.028 |
| LVIDs (mm) | 4.69 ± 0.22 | 4.70 ± 0.14 | 0.715 | 4.70 ± 0.57 | 2.66 ± 1.65* | 0.046 | 4.15 ± 0.80 | 2.40 ± 1.25* | 0.028 | 4.48 ± 0.45 | 1.23 ± 0.33*† | 0.028 |
| LVPWd (mm) | 1.39 ± 0.06 | 1.21 ± 0.12 | 0.144 | 1.62 ± 0.13 | 2.39 ± 0.68* | 0.046 | 1.53 ± 0.22 | 2.98 ± 0.54* | 0.028 | 1.47 ± 0.13 | 3.40 ± 0.47*† | 0.028 |
| LVd Mass (ASE, g) | 1.22 ± 0.07 | 1.17 ± 0.04 | 1.000 | 1.34 ± 0.10 | 1.42 ± 0.20* | 0.116 | 1.23 ± 0.06 | 1.48 ± 0.14* | 0.028 | 1.30 ± 0.12 | 1.37 ± 0.10* | 0.173 |
| EF (Teich, %) | 68.27 ± 2.03 | 69.32 ± 1.71 | 0.465 | 70.99 ± 7.47 | 85.77 ± 15.88 | 0.046 | 76.80 ± 9.55 | 78.64 ± 15.76* | 0.753 | 71.27 ± 5.93 | 93.58 ± 2.79* | 0.028 |
| FS (%) | 33.85 ± 1.66 | 34.56 ± 1.25 | 0.465 | 36.26 ± 5.98 | 55.47 ± 18.05 | 0.046 | 41.75 ± 9.17 | 45.70 ± 16.33* | 0.600 | 36.41 ± 4.87 | 62.09 ± 5.24* | 0.028 |
| EDV (Teich, mL) | 0.81 ± 0.13 | 0.84 ± 0.54 | 0.273 | 0.90 ± 0.10 | 0.48 ± 0.29 | 0.046 | 0.81 ± 0.15 | 0.21 ± 0.13* | 0.027 | 0.80 ± 0.15 | 0.09 ± 0.03*† | 0.028 |
| ESV (Teich, mL) | 0.25 ± 0.03 | 0.26 ± 0.02 | 0.715 | 0.26 ± 0.09 | 0.10 ± 0.15 | 0.046 | 0.19 ± 0.09 | 0.06 ± 0.06* | 0.028 | 0.23 ± 0.06 | 0.01 ± 0.00* | 0.028 |
| SV (Teich, mL) | 0.56 ± 0.10 | 0.58 ± 0.04 | 0.273 | 0.63 ± 0.07 | 0.38 ± 0.17* | 0.028 | 0.62 ± 0.11 | 0.15 ± 0.07* | 0.028 | 0.57 ± 0.12 | 0.08 ± 0.03*† | 0.028 |
| CO (Teich, ml/min) | 196.50 ± 21.04 | 217.09 ± 32.77 | 0.144 | 202.75 ± 30.09 | 180.98 ± 80.83 | 0.600 | 191.91 ± 30.53 | 88.93 ± 56.02* | 0.028 | 184.78 ± 31.10 | 38.14 ± 20.91*† | 0.028 |
| MV E Vel (m/s) | 0.73 ± 0.09 | 0.80 ± 0.11 | 0.144 | 0.77 ± 0.04 | 0.57 ± 0.05* | 0.028 | 0.78 ± 0.08 | 0.37 ± 0.07*† | 0.028 | 0.74 ± 0.06 | 0.33 ± 0.06*† | 0.028 |
| MV A Vel (m/s) | 0.57 ± 0.18 | 0.71 ± 0.11 | 0.144 | 0.47 ± 0.14 | 0.90 ± 0.09 | 0.028 | 0.46 ± 0.06 | 0.74 ± 0.19 | 0.028 | 0.63 ± 0.14 | 0.69 ± 0.19 | 0.173 |
| MV E/A Ratio | 1.38 ± 0.48 | 1.15 ± 0.35 | 0.715 | 1.77 ± 0.50 | 0.64 ± 0.10* | 0.028 | 1.65 ± 0.16 | 0.50 ± 0.05*† | 0.028 | 1.24 ± 0.36 | 0.49 ± 0.08*† | 0.028 |

Data are expressed as means ± SD; CO, cardiac output; E, epinephrine; E+NE, epinephrine and norepinephrine; EDV, end-diastolic volume; EF, ejection fraction; ESV, end-systolic volume; FS, fractional shortening; HR, heart rate; IVSd, interventricular septum thickness at end –diastole; LVd Mass, the left ventricular mass at end diastole; LVIDd, left ventricular internal dimension at end –diastole; LVIDs, left ventricular internal dimension at end –systole; LVPWd, left ventricular posterior wall thickness at end –diastole; MV A vel, peak late diastolic mitral valve velocity; MV E vel, peak early diastolic mitral valve velocity; NE, norepinephrine; sham, 0.9% saline; SV, stroke volume. *$p < 0.05$ vs. sham group. †$p$ <0.05 vs. E group.
